# Temperature-Dependent Regulation of Proteostasis and Longevity

**DOI:** 10.3389/fragi.2022.853588

**Published:** 2022-03-24

**Authors:** Kavya Leo Vakkayil, Thorsten Hoppe

**Affiliations:** ^1^ Institute for Genetics and Cologne Excellence Cluster on Cellular Stress Responses in Aging-Associated Diseases (CECAD), University of Cologne, Cologne, Germany; ^2^ Center for Molecular Medicine Cologne (CMMC), University of Cologne, Cologne, Germany

**Keywords:** temperature, proteostasis, protein aggregation, longevity, *Caenorhabditis elegans*, thermosensation, AFD neuron, cell non-autonomous signaling

## Abstract

Temperature is an important environmental condition that determines the physiology and behavior of all organisms. Animals use different response strategies to adapt and survive fluctuations in ambient temperature. The hermaphrodite *Caenorhabditis elegans* has a well-studied neuronal network consisting of 302 neurons. The bilateral AFD neurons are the primary thermosensory neurons in the nematode. In addition to regulating thermosensitivity, AFD neurons also coordinate cellular stress responses through systemic mechanisms involving neuroendocrine signaling. Recent studies have examined the effects of temperature on altering various signaling pathways through specific gene expression programs that promote stress resistance and longevity. These studies challenge the proposed theories of temperature-dependent regulation of aging as a passive thermodynamic process. Instead, they provide evidence that aging is a well-defined genetic program. Loss of protein homeostasis (proteostasis) is one of the key hallmarks of aging. Indeed, proteostasis pathways, such as the heat shock response and aggregation of metastable proteins, are also controlled by thermosensory neurons in *C. elegans*. Prolonged heat stress is thought to play a critical role in the development of neurodegenerative protein misfolding diseases in humans. This review presents the latest evidence on how temperature coordinates proteostasis and aging. It also discusses how studies of poikilothermic organisms can be applied to vertebrates and provides new therapeutic strategies for human disease.

## Introduction

“Homeostasis”—a term coined by Walter Cannon—is the self-regulating dynamic process by which an organism maintains internal stability in response to external conditions (Billman, 2020). Regulation of core body temperature (thermoregulation) is one of the ways to maintain this balance. Homeotherms (warm-blooded animals) such as mammals and birds keep a constant core temperature via hypothalamic regulation of heat production and dissipation. In contrast, poikilotherms (cold-blooded animals) such as flies, nematodes, amphibians, and reptiles lack this thermoregulatory ability. As a result, the core temperature of poikilothermic organisms fluctuates with ambient temperature changes (Tabarean et al., 2010). Another necessary means of maintaining a constant internal milieu is to preserve the integrity of cellular macromolecules such as proteins and maintain a balanced cellular proteome. Protein homeostasis (proteostasis) is promoted by a network of cellular quality control pathways (Kaushik and Cuervo, 2015), including molecular chaperones, the ubiquitin–proteasome system (UPS), and the autophagy machinery (Chen et al., 2011). Regulated synthesis of polypeptides involves proper protein folding by molecular chaperones, whereas the selective degradation of damaged proteins is mediated by the UPS and the autophagy machinery (Hoppe and Cohen, 2020).

Endogenous and exogenous challenges constantly threaten the integrity of the organismal proteome and affect longevity (Gumeni et al., 2017). Cells cope with cellular stress by adopting sophisticated protective measures. For example, to combat heat stress, organisms have an ancient, highly conserved genetic program termed the heat shock response (HSR) (Morimoto, 1998). An abnormal rise in temperature that triggers protein misfolding and aggregation activates the heat shock transcription factor-1 (HSF-1) in the cytoplasm. Subsequently, HSF-1 converts from an inactive monomer to an active trimeric form and activates expression of heat shock proteins (HSPs), including molecular chaperones (Anckar and Sistonen, 2011). The suppression of HSR in early adulthood renders organisms susceptible to various environmental stressors and eventually leads to breakdown of proteostasis (Ben-Zvi et al., 2009; Labbadia and Morimoto, 2015). The capacity of the proteostasis network (PN) decreases with age, leading to the accumulation of damaged proteins and chronic age-related diseases (Balchin et al., 2016). This review summarizes how proteostasis and longevity are modulated by changes in environmental temperature, focusing on studies in *Caenorhabditis elegans*.

## The Influence of Temperature on Proteostasis

The soil-dwelling nematode *C. elegans* is a poikilothermic organism that must constantly adapt its physiology to the changing temperature conditions in its natural habitat (Mendenhall et al., 2017). The worm perceives environmental temperature by the thermosensory neurons named AFD, AWC, ASI, and ASJ (Ohta et al., 2014). The interneurons AIY and AIZ receive thermal inputs from upstream thermosensory neurons, which are further integrated by the RIA interneurons (Kimata et al., 2012). The bilateral AFD neuron is the primary thermosensory neuron that senses ambient temperatures to regulate animal behavior (Goodman and Sengupta, 2018). Thermosensation by AFD and AIY neurons has been associated with organismal proteostasis, particularly in the regulation of the HSR. The heat shock response has always been considered a cell-autonomous response triggered by accumulation of damaged proteins. Surprisingly, a pioneering study by the Morimoto laboratory shows that thermosensory neurons control the heat shock response of somatic tissues. Consequently, worms lacking these neurons exhibit reduced thermotolerance when exposed to increased temperature (Prahlad et al., 2008). The temperature-dependent activation of AFD neurons triggers the HSR in distal tissues by serotonin signaling (Tatum et al., 2015). Reduced HSR in thermosensory mutants suggests a potential increase in protein misfolding. However, the data prove otherwise. At physiological temperatures (20°C), thermosensory mutants suppress protein aggregation and toxicity in multiple tissues. This counterintuitive result shows that the neuronal circuitry based on the thermosensory AFD neurons differentiates between acute heat stress and chronic protein misfolding stress (Prahlad and Morimoto, 2011).

Several interesting observations have been made in recent years despite the lack of mechanistic insight into the effects of temperature on proteostasis. For example, when late larval stages of *C. elegans* grown at 20°C are exposed to 25°C for 1 day, several stress responses are regulated. The unfolded protein response in the endoplasmic reticulum (UPR^ER^) is strongly activated, whereas HSR increases only transiently after a 1-day exposure to 25°C. Surprisingly, a change in ambient temperature from 20 to 25°C can affect selective protein degradation via the UPS. UPS activity increases in the intestine but not in muscle cells, suggesting tissue-specific regulation of protein degradation (Pispa et al., 2020). Increasing temperature conditions also modulates mRNA translation. One of the five eukaryotic initiation factor (eIF)-4E proteins in *C. elegans*, IFE-2, increases the translational efficiency of certain mRNAs at 25°C (Song et al., 2010). Furthermore, hormetic heat shock of 1 h at 36°C induces autophagy and selective HSR in adult worms, associated with decreased protein aggregation (Kumsta et al., 2017). These studies suggest that *C. elegans* respond to elevated temperature conditions not only by triggering HSR but also other branches of the PN, including translation, protein degradation, and UPR.

**FIGURE 1:**
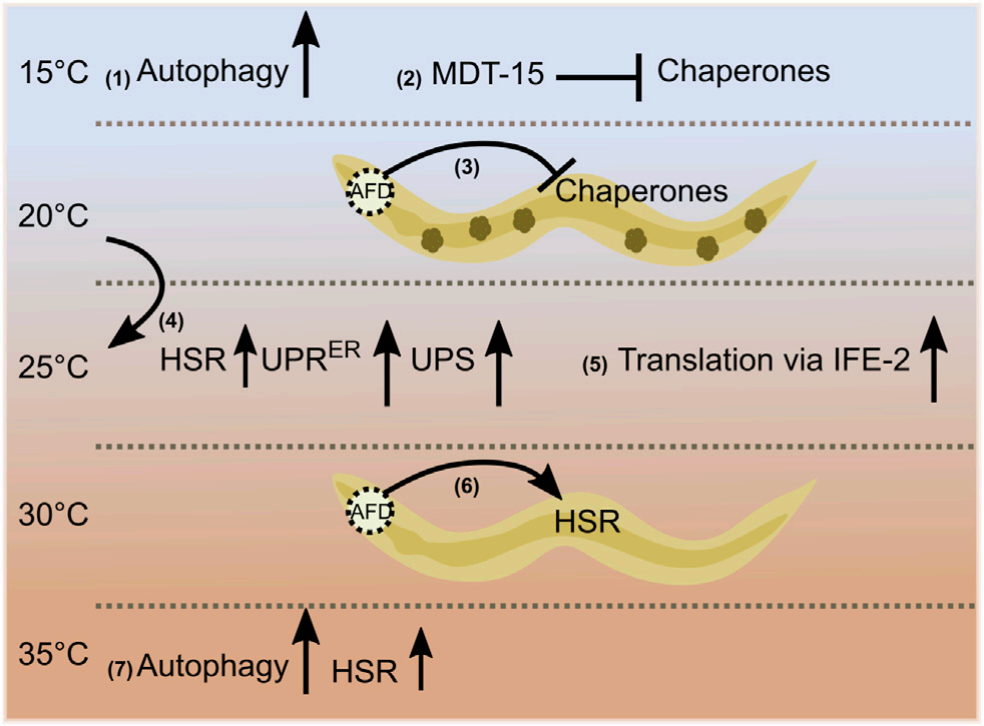
Temperature-dependent regulation of proteostasis. Autophagy and chaperone levels are affected at low temperatures via fatty acid signaling (1), (2). A wild-type thermosensory circuit attenuates protein folding during chronic stress; small dark green structures inside the worm indicate protein aggregates (3). A 1-day transfer of late larval staged *C. elegans* from 20 to 25°C increases the heat shock response [HSR (mildly, small arrow)], the unfolded protein response in the endoplasmic reticulum (UPR^ER^), and the activity of the ubiquitin/proteasome-system (UPS) in the intestine (4). The translational efficiency of selective mRNAs (*msh-4/him-14*, *msh-5*) increases at 25°C via an eIF4E family protein, IFE-2 (5). AFD thermosensory neuron cells non-autonomously regulate the heat shock response [acute heat shock (HS) of 30°C for 15 min] (6). Hormetic heat shock of 1 h at 36°C induces autophagy and selective HSR (7).

Low temperatures regulate proteostasis pathways via modulation of lipid homeostasis. Culturing worms at 15°C promotes autophagy through signaling mediated by the adiponectin receptor PAQR-2. PAQR-2 increases fatty acid desaturase FAT-7, which triggers the biosynthesis of polyunsaturated fatty acids, namely γ-linolenic acid and arachidonic acid, to induce autophagy (Chen et al., 2019). Mediator complex subunit—mediator 15 (MDT-15/MED15) regulates the expression of *fat-7* at lower temperatures. Decreasing MDT-15 levels at 15°C increases protein aggregates and cytosolic chaperone expression via HSF-1 as an adaptive response (Dongyeop Lee et al., 2019). Alternatively, gene ontology analysis shows that genes regulated by cold warming—exposure to cold shock (4°C) followed by recovery at normal temperatures (20°C)—are involved in biological processes such as autophagy and proteostasis (Jiang et al., 2018). These studies highlight the importance of different temperature conditions in regulating proteome dynamics (Figure 1). The maintenance of proteostasis is essential for healthy aging, and its impairment is considered one of the critical hallmarks of aging (López-Otín et al., 2013). In the following section, studies on the influence of temperature on longevity are described in detail.

## Modulation of Organismal Longevity by Temperature

Poikilothermic animals have shorter lifespans at higher temperatures than at lower temperatures (Lamb, 1968). *C. elegans*, for example, has a mean lifespan of 15.2 ± 0.5 (mean lifespan ± SEM provided) and 26.1 ± 0.6 days at 25 and 15°C, respectively (Lee and Kenyon, 2009). Pioneering studies led to the postulation of two theories, rate-of-living theory and threshold theory, to explain the effects of temperature on lifespan. Pearl’s rate-of-living theory attempts to determine lifespan as a simple rate-limiting response and proposes that the rate of aging increases at higher temperatures (Shaw and Bercaw, 1962). Alternatively, the threshold theory introduces two phases that determine lifespan—aging and dying. It states that the rate of aging is independent of temperature, while the rate of dying depends on temperature (Smith, 1963). Interestingly, the effect of temperature on lifespan has been considered mainly as a passive thermodynamic process in the aforementioned scenarios. However, several studies on poikilothermic organisms, including *C. elegans*, provide considerable evidence to the contrary.

Sensory perception of thermal signals via thermosensory neurons plays a critical role in regulating *C. elegans* lifespan. AFD thermosensory neurons control lifespan at warm temperatures via a steroid signaling pathway. If AFD neuron function is knocked down from early development by genetic mutation or laser ablation, lifespan shortens at 25°C but not at 15°C. Thermosensation via these neurons contributes to inhibition of the nuclear hormone receptor (NHR) DAF-12 via DAF-9/Cytochrome P450 (Lee and Kenyon, 2009). Neuronal synaptic transmission may also affect longevity at 25°C (He et al., 2009). The cyclic AMP (cAMP)-responsive element binding protein, CRH-1/CREB, increases expression of the FMRFamide-like neuropeptide FLP-6 in AFD neurons, promoting adult lifespan. Increased expression of FLP-6 increases DAF-9/sterol signaling in AIY neurons and downregulates insulin signaling in the gut to regulate longevity (Chen et al., 2016). Heat-sensitive ASJ neurons, when ablated, extend lifespan at 25°C. ASJ neurons act via UNC-31-dependent release of two neuropeptides, INS-6 and DAF-28, to inhibit the FOXO transcription factor DAF-16 in the gut (Zhang et al., 2018). STR-2, a G-protein-coupled receptor expressed in AWC^ON^ and ASI neurons, controls lifespan at 20°C and higher temperatures. STR-2 fine-tunes neutral lipid levels in non-neuronal tissues to adapt to higher temperatures to maintain lifespan (Dixit et al., 2020). Systemic temperature signaling at culture temperatures (17–23°C) occurs via HSF-1 activity in non-neuronal cells such as the gut or muscle. Downstream signaling modifies the thermotaxis circuit via the nuclear hormone receptor NHR-69-mediated estrogen signaling (Sugi et al., 2011). HSF-1 not only regulates thermotaxic behavioral performance but also contributes to extending animal lifespan at warm temperatures. Pioneering work by Lee and Kenyon showed that a reduction in HSF-1 function further shortened lifespan at 22.5°C. Consistent with this, recent data show that overexpression of *hsf-1* in neurons protects animals at 25°C and extends their lifespan (Chauve et al., 2021). Overexpression of the proteasomal subunit *rpn-6.1* extends lifespan independently of HSF-1 in a DAF-16-dependent manner at 25°C (Vilchez et al., 2012). Temperature experiences under different growth conditions can lead to different outcomes via the same central player. For example, the co-chaperone DAF-41/p23 modulates lifespan in different ways at warm and cold temperatures, as *daf-41* mutants live longer at higher temperatures but are short-lived at cold temperatures (Horikawa et al., 2015).

**FIGURE 2:**
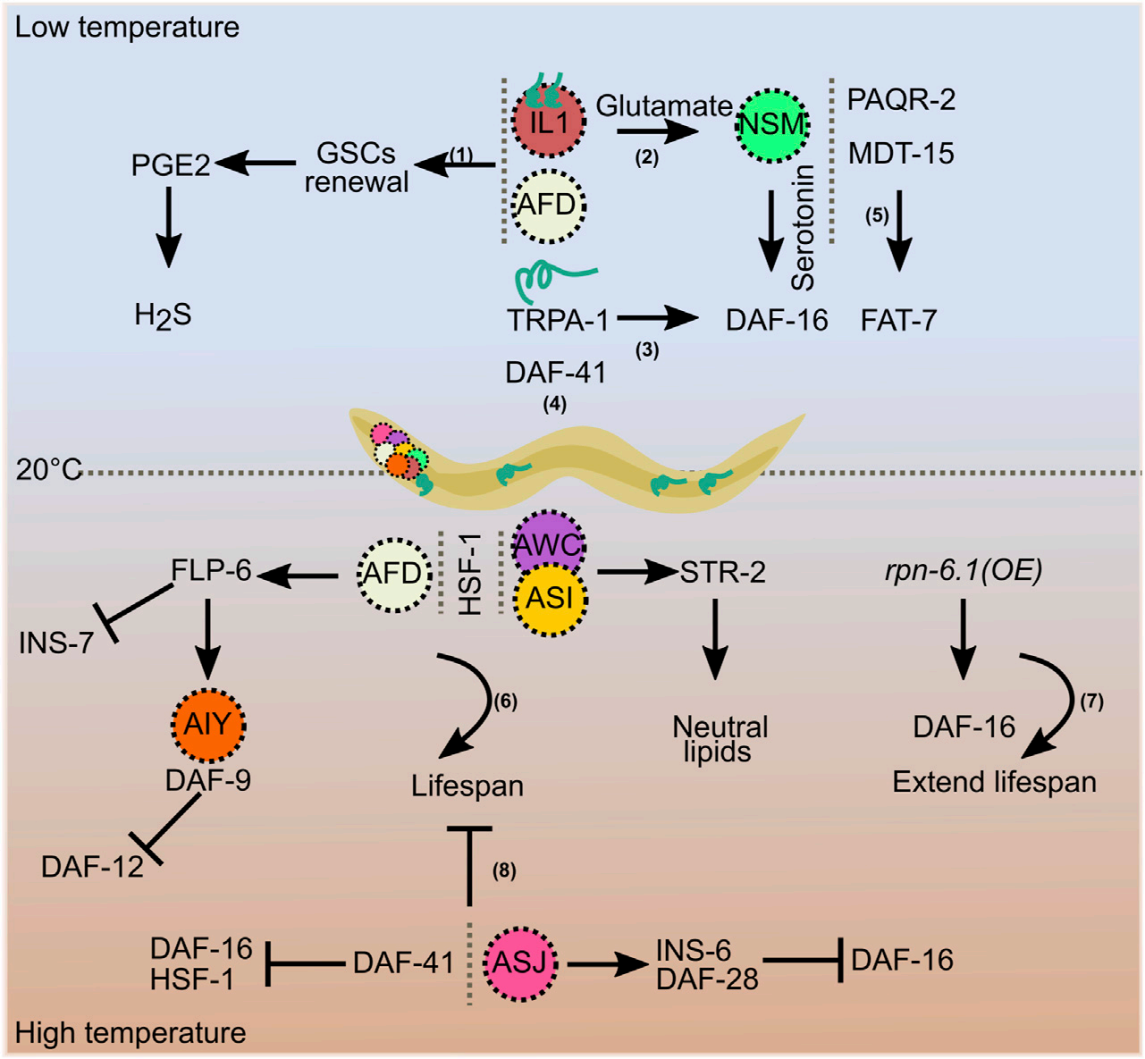
Temperature-dependent regulation of longevity. Increased lifespan at low temperatures is regulated by signals involving germline stem cells (1), AFD and IL1 neurons (1 and 2), TRPA-1 (3), the co-chaperone DAF-41 (4), and fatty acids (5). AFD, AWC, and ASI neurons, and HSF-1 maintain lifespan at higher temperatures (6), overexpression of *rpn-6.1* prolongs it (7), and ASJ neurons and DAF-41 shorten lifespan at higher temperatures (8).

Temperature sensing across different tissues and lipid signaling interact to regulate lifespan at cold temperatures. Low temperatures significantly extend *C. elegans* lifespan via the cold-sensitive transient receptor potential (TRP) channel, TRPA-1. TRPA-1 is a non-selective cation channel that is also permeable to calcium. Expression of TRPA-1 in neurons or intestine promotes DAF-16 activity via a genetic program involving a calcium-sensitive kinase, PKC-2, and a DAF-16 kinase, SGK-1. Intriguingly, this study shows that the intestine, which is a non-excitable tissue in worms, functions as a cold receptor (Xiao et al., 2013). TRPA-1 acts in the cold-sensitive IL1 sensory neurons. Glutamatergic and serotoninergic signals from IL1 and NSM neurons, respectively, activate a pro-longevity cascade. This neuroendocrine signaling regulates DAF-16 function in intestinal cells (Zhang et al., 2018). Moreover, temperature signaling from IL1 and AFD neurons maintains germline proliferation and delays germline stem cell (GSC) exhaustion. Prostaglandin E2 (PGE2) signals from adult GSCs communicate with the gut to produce hydrogen sulfide (H_2_S). Thus, germline and somatic tissues contribute to cold-induced longevity (Hyun Ju Lee et al., 2019). PAQR-2, MDT-15, and azelaic acid (AzA) support longevity at low temperatures through fatty acid–mediated signaling (Chen et al., 2019; Dongyeop Lee et al., 2019; Bai et al., 2021). Although these different factors regulating life expectancy in warm and cold temperatures have been identified, it remains unclear how they communicate with each other and coordinate their functions. Extensive studies have helped to identify the main players that regulate longevity at cold and warm temperatures. Cultivation at low temperatures is beneficial, while warm temperatures affect longevity. However, this relationship does not always hold. When *C. elegans* is exposed to high temperatures during early developmental stages, adult lifespan increases (Zhang et al., 2015). This transient heat stress during early life activates long-lasting defense responses via histone acetyltransferase CBP-1 and the chromatin remodeling complex SWI/SNF, which promote longevity (Zhou et al., 2019). These studies show that the temperature-dependent effects on aging are a well-regulated event controlled by genetic and epigenetic factors (Figure 2).

## Discussion

The evidence discussed thus far reveals a common theme in the temperature-dependent regulation of proteostasis and longevity. Environmental signals such as temperature influence organismal physiology via non-autonomous cell signaling mechanisms. Neurons act as receptors for thermal information and send signals mediated by small molecules to distal tissues. Insulin-like peptides, FMRFamide-like peptides, biogenic amines, and neurotransmitters are critical for triggering downstream responses.

Studies in poikilothermic animals have undoubtedly improved our understanding of temperature-dependent effects on organismal survival. However, many questions remain unanswered, particularly regarding the regulation of proteostasis. AFD thermosensory neurons and associated neuroendocrine signaling are well studied with respect to HSR. Surprisingly, there is little evidence of molecular players controlling other proteostasis pathways in response to temperature. In particular, it is unclear how temperature affects the UPS, the autophagy machinery, the unfolded protein response, and the genetic components involved. In addition to AFD, AWC, ASI, and ASJ neurons, the intestine act as temperature sensors in *C. elegans*. How thermosensation mediates proteostasis via these neurons and the gut remains to be elucidated. A crucial step would be to analyze how cell-specific thermosensory receptors and circuits control organismal proteostasis. *C. elegans* adapts to different environmental temperatures by calibrating unsaturated fatty acid levels to maintain optimal membrane fluidity (Ma et al., 2015). The role of fatty acids in regulating distinct proteostasis pathways under different temperature conditions is still unexplored. Further studies are needed to clarify these crucial issues.

## Temperature—A Potential Therapeutic Intervention?

There are few studies demonstrating the effects of temperature on proteostasis and homeotherm longevity. However, some basic principles remain. Temperature determines, in part, the effects of birth time on human fetal development and longevity. In particular, an increase in ambient temperature at birth has detrimental effects (Flouris et al., 2009). The central thermostat in the preoptic area controls core body temperature (CBT) in homeotherms. Transgenic mice overexpressing the mitochondrial membrane protein uncoupling protein 2 (UCP2) in hypocretin neurons (Hcrt-UCP2 mice) exhibit increased hypothalamic temperature and reduced CBT. A modest but sustained reduction in CBT increases the life expectancy of Hcrt-UCP2 mice (Conti et al., 2006). Further studies have also shown that gonad-dependent differences in CBT affect life expectancy in a sex-specific manner (Sanchez-Alavez et al., 2011). In addition, selective temperature conditions may alter the pathophysiology of age-related diseases. Aggregation of damaged proteins contributes to neurodegenerative diseases. A recent review suggested heat therapy that promotes chaperone expression as a potential treatment strategy (Hunt et al., 2020). Further evidence suggests that sauna bathing—a passive heat therapy—reduces the risk of death from cardiovascular disease (Laukkanen et al., 2018). Swimming in cold water (temperature <5°C) is also beneficial for experienced healthy individuals when practiced with caution (Knechtle et al., 2020). Cold-shock proteins such as RNA-binding motif protein 3, RBM3, are essential for maintaining synapses in laboratory mouse models of neurodegenerative diseases. These results suggest a potential role for therapeutic human hypothermia in achieving neuroprotective effects (Peretti et al., 2015). In-depth analyses in higher-order animals may help to exploit the benefits of temperature in improving organismal health. On a positive note, previous results from a poikilothermic animal may serve as a springboard for exploring this potential therapeutic area.
